# Supplementary material for: Identification of photoperiod-induced specific miRNAs in the adrenal glands of Sunite sheep (Ovis aries)
Source: Front Vet Sci. 2022 Jul 22;9:888207. doi: 10.3389/fvets.2022.888207 (PMC9354845; doi:10.3389/fvets.2022.888207)
Supplement: Supplementary file 1 [file Data_Sheet_1.ZIP › Supplementary file/miRDeep2 file.zip!/miRDeep2 file/Know_Structure/SPLP42A2/oar-mir-25.pdf]

miRBase precursor : oar-mir-25
Total read count : 21226
oar-miR-25 read count : 21135
remaining reads : 91

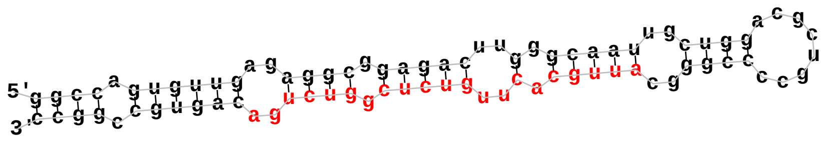

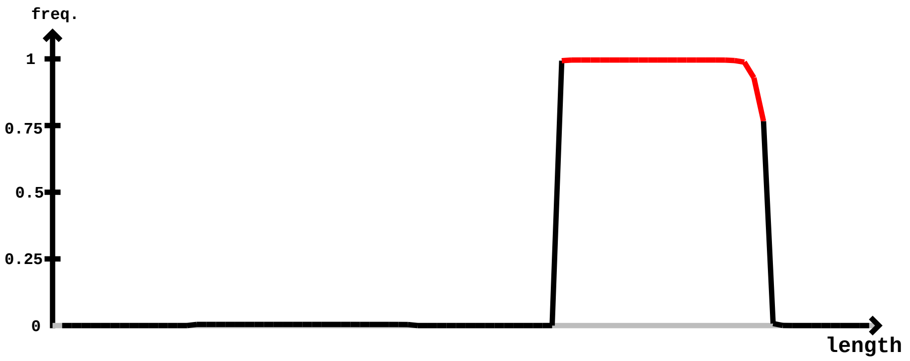


```
                                                oar-miR-25
5'- ggccaguguugagaggcggagacuugggcaauugcuggacgcugccccgggcauugcacuugucucggucugacagugccggcc   -3'    exp
    ((((.((((((..((((.(((((..(.(((((..((((........))))..))))).)..))))).)))).)))))).))))   reads  mm  sample
    .............aggcggagacuugggca......................................................   1      0   seq
    .............aggcggagacuugggcaauu...................................................   1      0   seq
    .............aggcggagacuugggcaauug..................................................   1      0   seq
    .............aggcggagacuugggcaauugc.................................................   8      0   seq
    .............aggcggagacuugggcaauugcu................................................   80     0   seq
    ..................................................gcauugcacuugucucggucug............   2      0   seq
    ...................................................cauugcacuugucucgg................   5      0   seq
    ...................................................cauugcacuugucucggu...............   38     0   seq
    ...................................................cauugcacuugucucgguc..............   124    0   seq
    ...................................................cauugcacuugucucggucu.............   1241   0   seq
    ...................................................cauugcacuugucucggucug............   3451   0   seq
    ...................................................cauugcacuugucucggucuga...........   16060  0   seq
    ...................................................cauugcacuugucucggucugac..........   153    0   seq
    ...................................................cauugcacuugucucggucugaca.........   8      0   seq
    ....................................................auugcacuugucucgguc..............   1      0   seq
    ....................................................auugcacuugucucggucu.............   3      0   seq
    ....................................................auugcacuugucucggucug............   7      0   seq
    ....................................................auugcacuugucucggucuga...........   40     0   seq
    .....................................................uugcacuugucucggucuga...........   2      0   seq
```
